# TRANSFORMING DEMENTIA SERVICE DELIVERY: THE MENTAL HEALTH GERO-CHAMPIONS PROGRAM

**DOI:** 10.1093/geroni/igz038.1392

**Published:** 2019-11-08

**Authors:** Regina M Koepp

**Affiliations:** Atlanta VA Health Care System, Decatur, Georgia, United States

## Abstract

In order to provide effective mental health care to older adults with major neurocognitive disorders (e.g., Alzheimer’s disease and related dementias) within outpatient mental health clinics, mental health practitioners must possess a basic understanding of these disorders, the needs of and challenges faced by people living with dementia and their families, and effective treatment approaches for this population. The Mental Health Gero-Champions Program was established in 2015 at a large Veterans Affairs medical center with the aim of providing clinical support and opportunities for training to multidisciplinary mental health providers to enhance skills in assessing and treating older adults with neurocognitive disorders. This presentation will provide an overview of the Mental Health Gero-Champions Program, describe the development and implementation of this program, and discuss challenges and successes in sustaining this transformative initiative over time.

